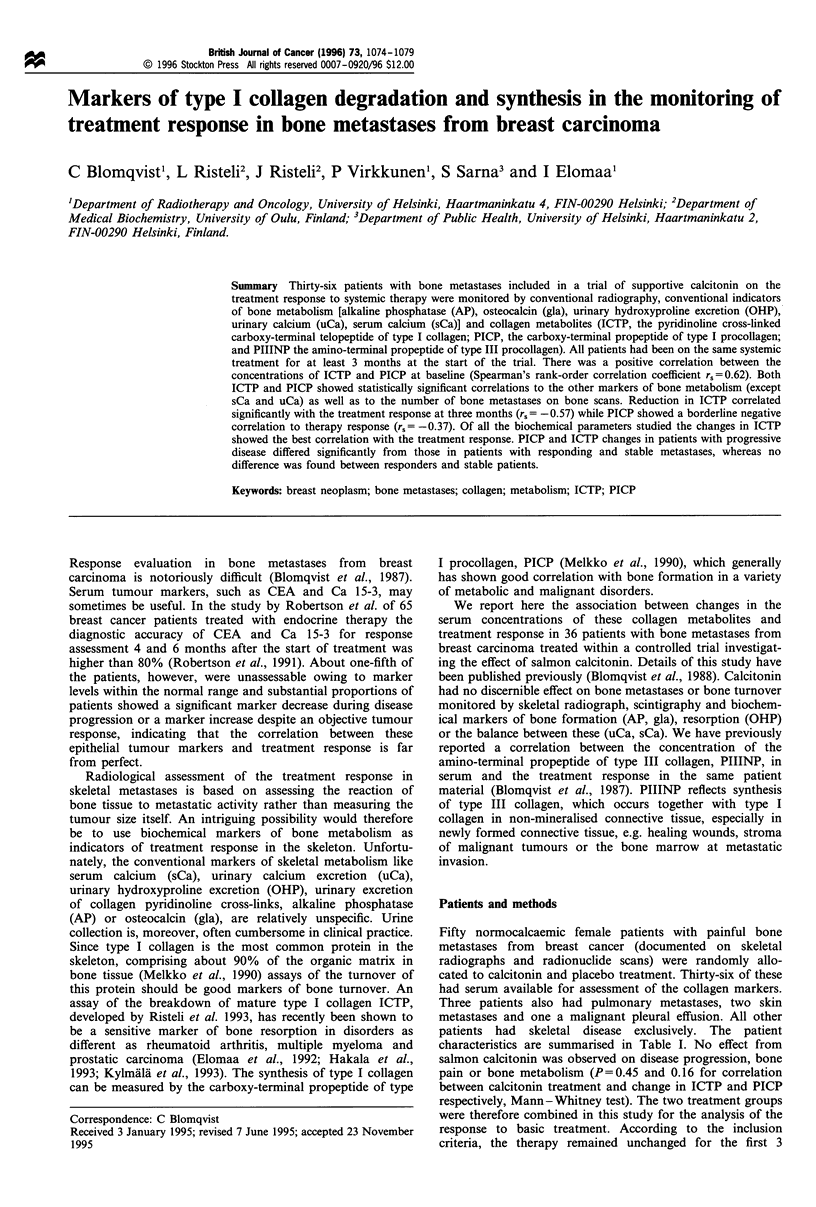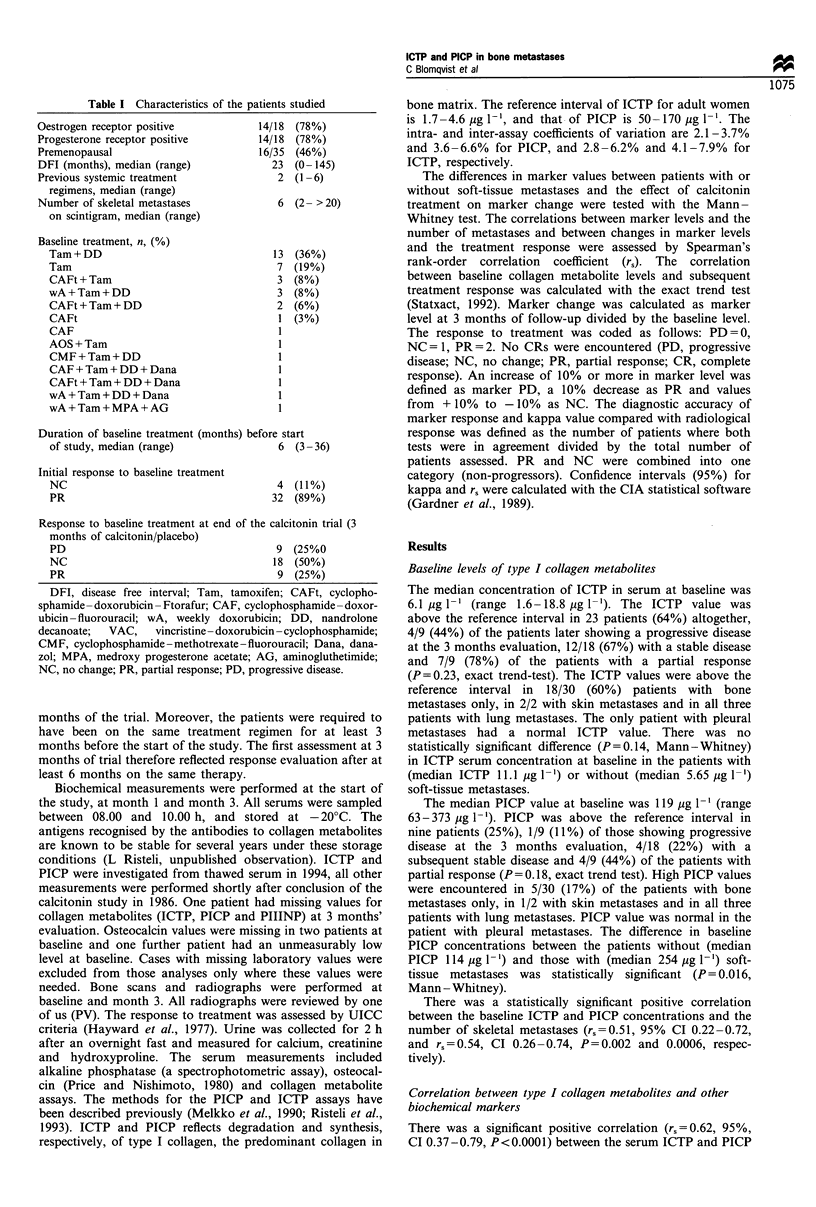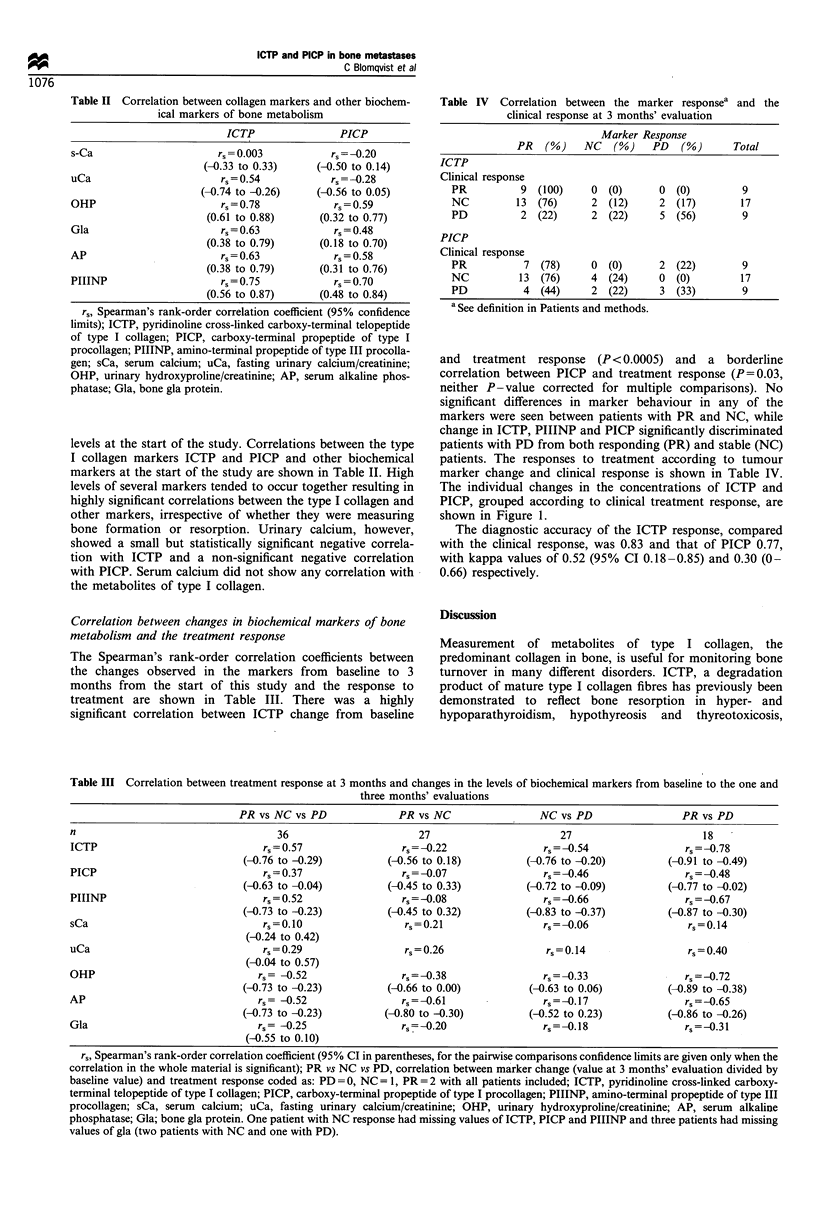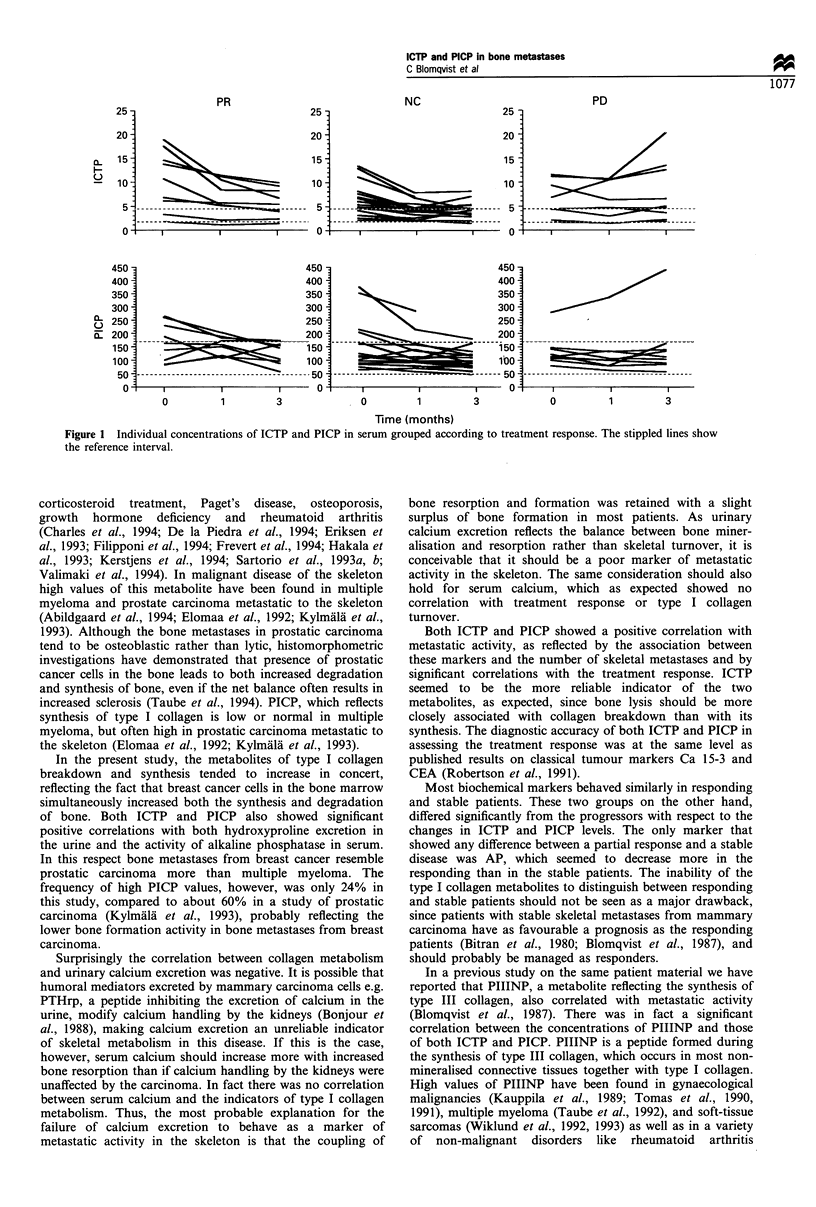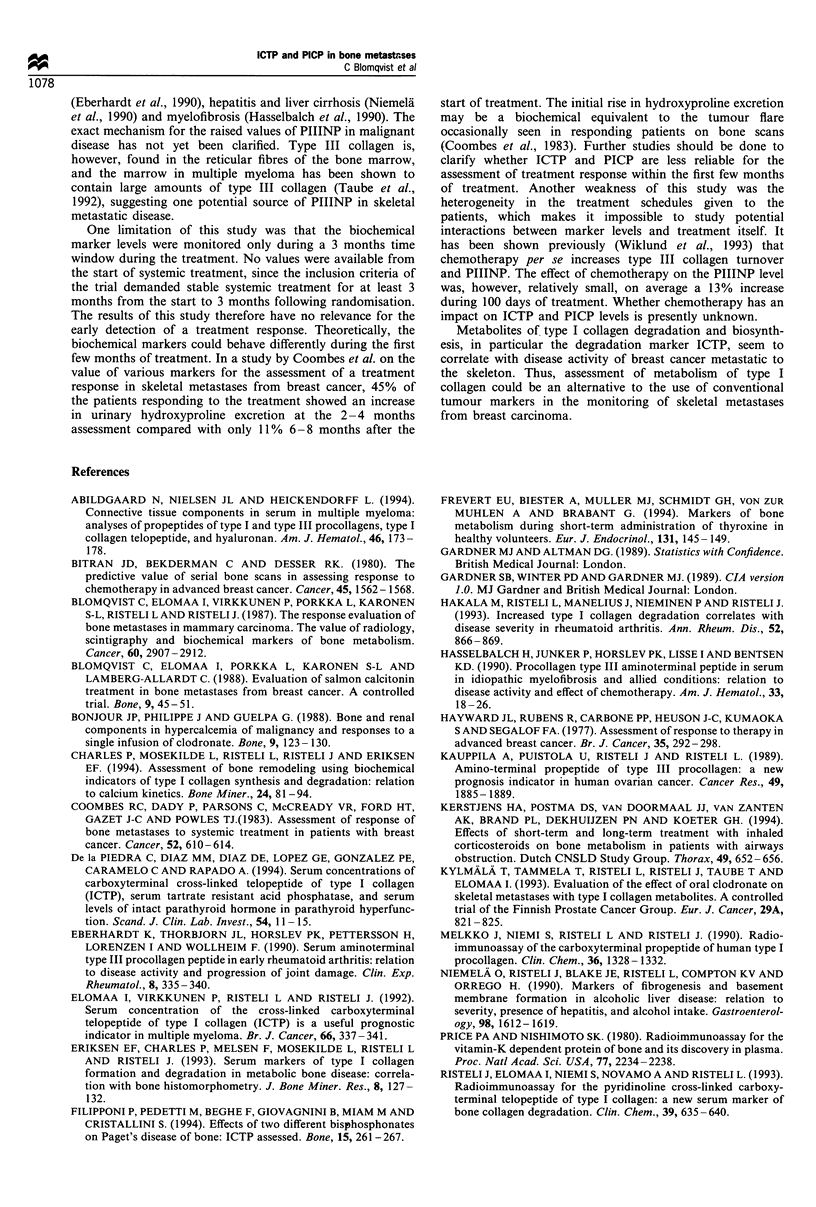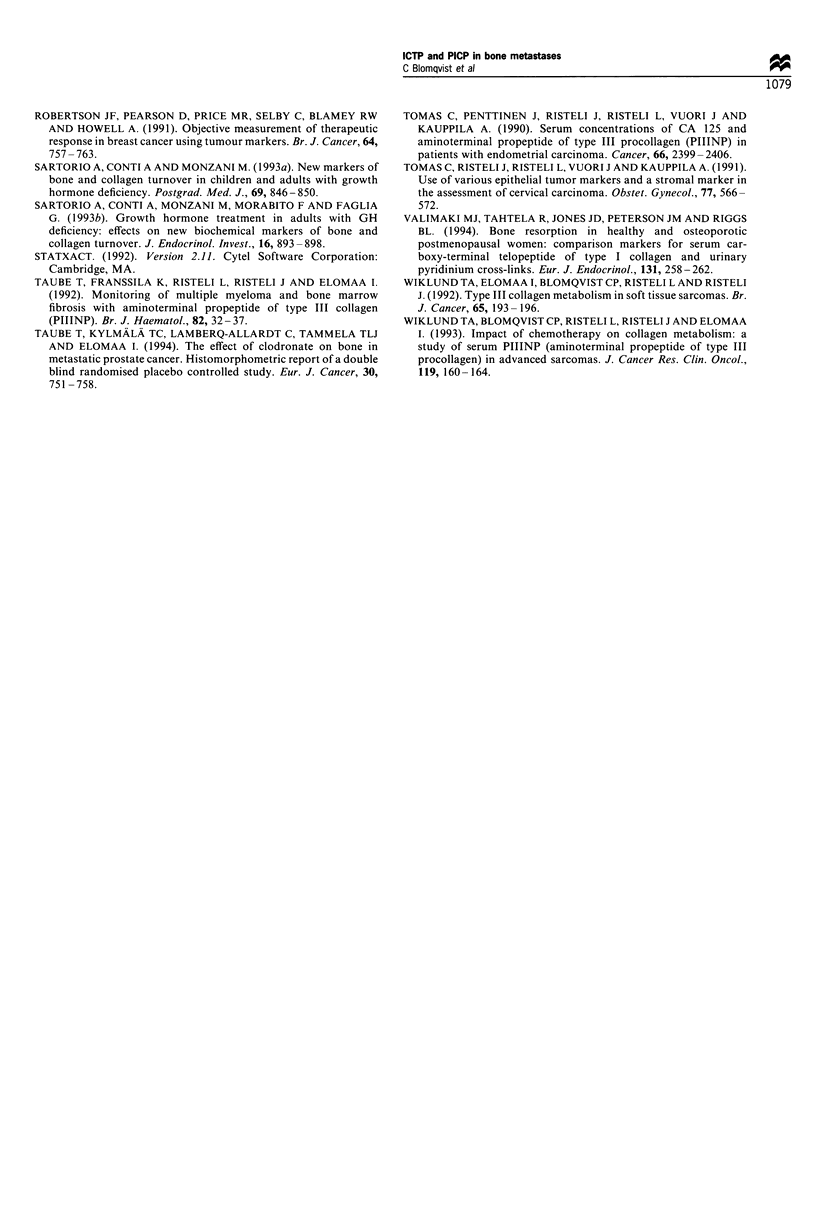# Markers of type I collagen degradation and synthesis in the monitoring of treatment response in bone metastases from breast carcinoma.

**DOI:** 10.1038/bjc.1996.207

**Published:** 1996-05

**Authors:** C. Blomqvist, L. Risteli, J. Risteli, P. Virkkunen, S. Sarna, I. Elomaa

**Affiliations:** Department of Radiotherapy and Oncology, University of Helsinki, Finland.

## Abstract

Thirty-six patients with bone metastases included in a trial of supportive calcitonin on the treatment response to systemic therapy were monitored by conventional radiography, conventional indicators of bone metabolism [alkaline phosphatase (AP), osteocalcin (gla), urinary hydroxyproline excretion (OHP), urinary calcium (uCa), serum calcium (sCa)] and collagen metabolites (ICTP, the pyridinoline cross-linked carboxy-terminal telopeptide of type I collagen; PICP, the carboxy-terminal propeptide of type I procollagen; and PIIINP the amino-terminal propeptide of type III procollagen). All patients had been on the same systemic treatment for at least 3 months at the start of the trial. There was a positive correlation between the concentrations of ICTP and PICP at baseline (Spearman's rank-order correlation coefficient rs = 0.62). Both ICTP and PICP showed statistically significant correlations to the other markers of bone metabolism (except sCa and uCa) as well as to the number of bone metastases on bone scans. Reduction in ICTP correlated significantly with the treatment response at three months (rs = - 0.57). while PICP showed a borderline negative correlation to therapy response (rs = - 0.37). Of all the biochemical parameters studied the changes in ICTP showed the best correlation with the treatment response. PICP and ICTP changes in patients with progressive disease differed significantly from those in patients with responding and stable metastases, whereas no difference was found between responders and stable patients.


British Journal of Cancer (1996) 73, 1074-1079
© 1996 Stockton Press All rights reserved 0007-0920/96 $12.00

### Markers of type I collagen degradation and synthesis in the monitoring of treatment response in bone metastases from breast carcinoma

C Blomqvist[1], L Risteli[2], J Risteli[2], P Virkkunen[1], S Sarna[3] and I Elomaa[1]

[1]*Department of Radiotherapy and Oncology, University of Helsinki, Haartmaninkatu 4, FIN-00290 Helsinki;* [2]*Department of Medical Biochemistry, University of Oulu, Finland;* [3]*Department of Public Health, University of Helsinki, Haartmaninkatu 2, FIN-00290 Helsinki, Finland.*

**Summary** Thirty-six patients with bone metastases included in a trial of supportive calcitonin on the treatment response to systemic therapy were monitored by conventional radiography, conventional indicators of bone metabolism [alkaline phosphatase (AP), osteocalcin (gla), urinary hydroxyproline excretion (OHP), urinary calcium (uCa), serum calcium (sCa)] and collagen metabolites (ICTP, the pyridinoline cross-linked carboxy-terminal telopeptide of type I collagen; PICP, the carboxy-terminal propeptide of type I procollagen; and PIIINP the amino-terminal propeptide of type III procollagen). All patients had been on the same systemic treatment for at least 3 months at the start of the trial. There was a positive correlation between the concentrations of ICTP and PICP at baseline (Spearman's rank-order correlation coefficient $r_s = 0.62$). Both ICTP and PICP showed statistically significant correlations to the other markers of bone metabolism (except sCa and uCa) as well as to the number of bone metastases on bone scans. Reduction in ICTP correlated significantly with the treatment response at three months ($r_s = -0.57$) while PICP showed a borderline negative correlation to therapy response ($r_s = -0.37$). Of all the biochemical parameters studied the changes in ICTP showed the best correlation with the treatment response. PICP and ICTP changes in patients with progressive disease differed significantly from those in patients with responding and stable metastases, whereas no difference was found between responders and stable patients.

**Keywords:** breast neoplasm; bone metastases; collagen; metabolism; ICTP; PICP

Response evaluation in bone metastases from breast carcinoma is notoriously difficult (Blomqvist *et al.*, 1987). Serum tumour markers, such as CEA and Ca 15-3, may sometimes be useful. In the study by Robertson *et al.* of 65 breast cancer patients treated with endocrine therapy the diagnostic accuracy of CEA and Ca 15-3 for response assessment 4 and 6 months after the start of treatment was higher than 80% (Robertson *et al.*, 1991). About one-fifth of the patients, however, were unassessable owing to marker levels within the normal range and substantial proportions of patients showed a significant marker decrease during disease progression or a marker increase despite an objective tumour response, indicating that the correlation between these epithelial tumour markers and treatment response is far from perfect.

Radiological assessment of the treatment response in skeletal metastases is based on assessing the reaction of bone tissue to metastatic activity rather than measuring the tumour size itself. An intriguing possibility would therefore be to use biochemical markers of bone metabolism as indicators of treatment response in the skeleton. Unfortunately, the conventional markers of skeletal metabolism like serum calcium (sCa), urinary calcium excretion (uCa), urinary hydroxyproline excretion (OHP), urinary excretion of collagen pyridinoline cross-links, alkaline phosphatase (AP) or osteocalcin (gla), are relatively unspecific. Urine collection is, moreover, often cumbersome in clinical practice. Since type I collagen is the most common protein in the skeleton, comprising about 90% of the organic matrix in bone tissue (Melkko *et al.*, 1990) assays of the turnover of this protein should be good markers of bone turnover. An assay of the breakdown of mature type I collagen ICTP, developed by Risteli *et al.* 1993, has recently been shown to be a sensitive marker of bone resorption in disorders as different as rheumatoid arthritis, multiple myeloma and prostatic carcinoma (Elomaa *et al.*, 1992; Hakala *et al.*, 1993; Kylmälä *et al.*, 1993). The synthesis of type I collagen can be measured by the carboxy-terminal propeptide of type I procollagen, PICP (Melkko *et al.*, 1990), which generally has shown good correlation with bone formation in a variety of metabolic and malignant disorders.

We report here the association between changes in the serum concentrations of these collagen metabolites and treatment response in 36 patients with bone metastases from breast carcinoma treated within a controlled trial investigating the effect of salmon calcitonin. Details of this study have been published previously (Blomqvist *et al.*, 1988). Calcitonin had no discernible effect on bone metastases or bone turnover monitored by skeletal radiograph, scintigraphy and biochemical markers of bone formation (AP, gla), resorption (OHP) or the balance between these (uCa, sCa). We have previously reported a correlation between the concentration of the amino-terminal propeptide of type III collagen, PIIINP, in serum and the treatment response in the same patient material (Blomqvist *et al.*, 1987). PIIINP reflects synthesis of type III collagen, which occurs together with type I collagen in non-mineralised connective tissue, especially in newly formed connective tissue, e.g. healing wounds, stroma of malignant tumours or the bone marrow at metastatic invasion.

### Patients and methods

Fifty normocalcaemic female patients with painful bone metastases from breast cancer (documented on skeletal radiographs and radionuclide scans) were randomly allocated to calcitonin and placebo treatment. Thirty-six of these had serum available for assessment of the collagen markers. Three patients also had pulmonary metastases, two skin metastases and one a malignant pleural effusion. All other patients had skeletal disease exclusively. The patient characteristics are summarised in Table I. No effect from salmon calcitonin was observed on disease progression, bone pain or bone metabolism ($P = 0.45$ and 0.16 for correlation between calcitonin treatment and change in ICTP and PICP respectively, Mann–Whitney test). The two treatment groups were therefore combined in this study for the analysis of the response to basic treatment. According to the inclusion criteria, the therapy remained unchanged for the first 3

Correspondence: C Blomqvist
Received 3 January 1995; revised 7 June 1995; accepted 23 November 1995

**Table I** Characteristics of the patients studied

| | | |
|---|---|---|
| Oestrogen receptor positive | 14/18 | (78%) |
| Progesterone receptor positive | 14/18 | (78%) |
| Premenopausal | 16/35 | (46%) |
| DFI (months), median (range) | 23 | (0-145) |
| Previous systemic treatment regimens, median (range) | 2 | (1-6) |
| Number of skeletal metastases on scintigram, median (range) | 6 | (2- >20) |
| Baseline treatment, *n*, (%) | | |
| Tam + DD | 13 | (36%) |
| Tam | 7 | (19%) |
| CAFt + Tam | 3 | (8%) |
| wA + Tam + DD | 3 | (8%) |
| CAFt + Tam + DD | 2 | (6%) |
| CAFt | 1 | (3%) |
| CAF | 1 | |
| AOS + Tam | 1 | |
| CMF + Tam + DD | 1 | |
| CAF + Tam + DD + Dana | 1 | |
| CAFt + Tam + DD + Dana | 1 | |
| wA + Tam + DD + Dana | 1 | |
| wA + Tam + MPA + AG | 1 | |
| Duration of baseline treatment (months) before start of study, median (range) | 6 | (3-36) |
| Initial response to baseline treatment | | |
| NC | 4 | (11%) |
| PR | 32 | (89%) |
| Response to baseline treatment at end of the calcitonin trial (3 months of calcitonin/placebo) | | |
| PD | 9 | (25%0 |
| NC | 18 | (50%) |
| PR | 9 | (25%) |

DFI, disease free interval; Tam, tamoxifen; CAFt, cyclophosphamide-doxorubicin-Ftorafur; CAF, cyclophosphamide-doxorubicin-fluorouracil; wA, weekly doxorubicin; DD, nandrolone decanoate; VAC, vincristine-doxorubicin-cyclophosphamide; CMF, cyclophosphamide-methotrexate-fluorouracil; Dana, danazol; MPA, medroxy progesterone acetate; AG, aminogluthetimide; NC, no change; PR, partial response; PD, progressive disease.

months of the trial. Moreover, the patients were required to have been on the same treatment regimen for at least 3 months before the start of the study. The first assessment at 3 months of trial therefore reflected response evaluation after at least 6 months on the same therapy.

Biochemical measurements were performed at the start of the study, at month 1 and month 3. All serums were sampled between 08.00 and 10.00 h, and stored at $-20°C$. The antigens recognised by the antibodies to collagen metabolites are known to be stable for several years under these storage conditions (L Risteli, unpublished observation). ICTP and PICP were investigated from thawed serum in 1994, all other measurements were performed shortly after conclusion of the calcitonin study in 1986. One patient had missing values for collagen metabolites (ICTP, PICP and PIIINP) at 3 months' evaluation. Osteocalcin values were missing in two patients at baseline and one further patient had an unmeasurably low level at baseline. Cases with missing laboratory values were excluded from those analyses only where these values were needed. Bone scans and radiographs were performed at baseline and month 3. All radiographs were reviewed by one of us (PV). The response to treatment was assessed by UICC criteria (Hayward *et al.*, 1977). Urine was collected for 2 h after an overnight fast and measured for calcium, creatinine and hydroxyproline. The serum measurements included alkaline phosphatase (a spectrophotometric assay), osteocalcin (Price and Nishimoto, 1980) and collagen metabolite assays. The methods for the PICP and ICTP assays have been described previously (Melkko *et al.*, 1990; Risteli *et al.*, 1993). ICTP and PICP reflects degradation and synthesis, respectively, of type I collagen, the predominant collagen in bone matrix. The reference interval of ICTP for adult women is 1.7-4.6 $\mu g\ l^{-1}$, and that of PICP is 50-170 $\mu g\ l^{-1}$. The intra- and inter-assay coefficients of variation are 2.1-3.7% and 3.6-6.6% for PICP, and 2.8-6.2% and 4.1-7.9% for ICTP, respectively.

The differences in marker values between patients with or without soft-tissue metastases and the effect of calcitonin treatment on marker change were tested with the Mann-Whitney test. The correlations between marker levels and the number of metastases and between changes in marker levels and the treatment response were assessed by Spearman's rank-order correlation coefficient ($r_s$). The correlation between baseline collagen metabolite levels and subsequent treatment response was calculated with the exact trend test (Statxact, 1992). Marker change was calculated as marker level at 3 months of follow-up divided by the baseline level. The response to treatment was coded as follows: PD = 0, NC = 1, PR = 2. No CRs were encountered (PD, progressive disease; NC, no change; PR, partial response; CR, complete response). An increase of 10% or more in marker level was defined as marker PD, a 10% decrease as PR and values from +10% to -10% as NC. The diagnostic accuracy of marker response and kappa value compared with radiological response was defined as the number of patients where both tests were in agreement divided by the total number of patients assessed. PR and NC were combined into one category (non-progressors). Confidence intervals (95%) for kappa and $r_s$ were calculated with the CIA statistical software (Gardner *et al.*, 1989).

### Results

### *Baseline levels of type I collagen metabolites*

The median concentration of ICTP in serum at baseline was 6.1 $\mu g\ l^{-1}$ (range 1.6-18.8 $\mu g\ l^{-1}$). The ICTP value was above the reference interval in 23 patients (64%) altogether, 4/9 (44%) of the patients later showing a progressive disease at the 3 months evaluation, 12/18 (67%) with a stable disease and 7/9 (78%) of the patients with a partial response ($P = 0.23$, exact trend-test). The ICTP values were above the reference interval in 18/30 (60%) patients with bone metastases only, in 2/2 with skin metastases and in all three patients with lung metastases. The only patient with pleural metastases had a normal ICTP value. There was no statistically significant difference ($P = 0.14$, Mann-Whitney) in ICTP serum concentration at baseline in the patients with (median ICTP 11.1 $\mu g\ l^{-1}$) or without (median 5.65 $\mu g\ l^{-1}$) soft-tissue metastases.

The median PICP value at baseline was 119 $\mu g\ l^{-1}$ (range 63-373 $\mu g\ l^{-1}$). PICP was above the reference interval in nine patients (25%), 1/9 (11%) of those showing progressive disease at the 3 months evaluation, 4/18 (22%) with a subsequent stable disease and 4/9 (44%) of the patients with partial response ($P = 0.18$, exact trend test). High PICP values were encountered in 5/30 (17%) of the patients with bone metastases only, in 1/2 with skin metastases and in all three patients with lung metastases. PICP value was normal in the patient with pleural metastases. The difference in baseline PICP concentrations between the patients without (median PICP 114 $\mu g\ l^{-1}$) and those with (median 254 $\mu g\ l^{-1}$) soft-tissue metastases was statistically significant ($P = 0.016$, Mann-Whitney).

There was a statistically significant positive correlation between the baseline ICTP and PICP concentrations and the number of skeletal metastases ($r_s = 0.51$, 95% CI 0.22-0.72, and $r_s = 0.54$, CI 0.26-0.74, $P = 0.002$ and 0.0006, respectively).

### *Correlation between type I collagen metabolites and other biochemical markers*

There was a significant positive correlation ($r_s = 0.62$, 95%, CI 0.37-0.79, $P < 0.0001$) between the serum ICTP and PICP

**Table II** Correlation between collagen markers and other biochemical markers of bone metabolism

| | *ICTP* | *PICP* |
|---|---|---|
| s-Ca | $r_s$=0.003 (–0.33 to 0.33) | $r_s$=–0.20 (–0.50 to 0.14) |
| uCa | $r_s$=0.54 (–0.74 to –0.26) | $r_s$=–0.28 (–0.56 to 0.05) |
| OHP | $r_s$=0.78 (0.61 to 0.88) | $r_s$=0.59 (0.32 to 0.77) |
| Gla | $r_s$=0.63 (0.38 to 0.79) | $r_s$=0.48 (0.18 to 0.70) |
| AP | $r_s$=0.63 (0.38 to 0.79) | $r_s$=0.58 (0.31 to 0.76) |
| PIIINP | $r_s$=0.75 (0.56 to 0.87) | $r_s$=0.70 (0.48 to 0.84) |

$r_s$, Spearman's rank-order correlation coefficient (95% confidence limits); ICTP, pyridinoline cross-linked carboxy-terminal telopeptide of type I collagen; PICP, carboxy-terminal propeptide of type I procollagen; PIIINP, amino-terminal propeptide of type III procollagen; sCa, serum calcium; uCa, fasting urinary calcium/creatinine; OHP, urinary hydroxyproline/creatinine; AP, serum alkaline phosphatase; Gla, bone gla protein.

levels at the start of the study. Correlations between the type I collagen markers ICTP and PICP and other biochemical markers at the start of the study are shown in Table II. High levels of several markers tended to occur together resulting in highly significant correlations between the type I collagen and other markers, irrespective of whether they were measuring bone formation or resorption. Urinary calcium, however, showed a small but statistically significant negative correlation with ICTP and a non-significant negative correlation with PICP. Serum calcium did not show any correlation with the metabolites of type I collagen.

*Correlation between changes in biochemical markers of bone metabolism and the treatment response*

The Spearman's rank-order correlation coefficients between the changes observed in the markers from baseline to 3 months from the start of this study and the response to treatment are shown in Table III. There was a highly significant correlation between ICTP change from baseline

**Table IV** Correlation between the marker response[a] and the clinical response at 3 months' evaluation

| | *Marker Response* | | | | | | |
|---|---|---|---|---|---|---|---|
| | *PR* | *(%)* | *NC* | *(%)* | *PD* | *(%)* | *Total* |
| *ICTP* | | | | | | | |
| Clinical response | | | | | | | |
| PR | 9 | (100) | 0 | (0) | 0 | (0) | 9 |
| NC | 13 | (76) | 2 | (12) | 2 | (17) | 17 |
| PD | 2 | (22) | 2 | (22) | 5 | (56) | 9 |
| *PICP* | | | | | | | |
| Clinical response | | | | | | | |
| PR | 7 | (78) | 0 | (0) | 2 | (22) | 9 |
| NC | 13 | (76) | 4 | (24) | 0 | (0) | 17 |
| PD | 4 | (44) | 2 | (22) | 3 | (33) | 9 |

[a] See definition in Patients and methods.

and treatment response ($P<0.0005$) and a borderline correlation between PICP and treatment response ($P=0.03$, neither $P$-value corrected for multiple comparisons). No significant differences in marker behaviour in any of the markers were seen between patients with PR and NC, while change in ICTP, PIIINP and PICP significantly discriminated patients with PD from both responding (PR) and stable (NC) patients. The responses to treatment according to tumour marker change and clinical response is shown in Table IV. The individual changes in the concentrations of ICTP and PICP, grouped according to clinical treatment response, are shown in Figure 1.

The diagnostic accuracy of the ICTP response, compared with the clinical response, was 0.83 and that of PICP 0.77, with kappa values of 0.52 (95% CI 0.18–0.85) and 0.30 (0–0.66) respectively.

### Discussion

Measurement of metabolites of type I collagen, the predominant collagen in bone, is useful for monitoring bone turnover in many different disorders. ICTP, a degradation product of mature type I collagen fibres has previously been demonstrated to reflect bone resorption in hyper- and hypoparathyroidism, hypothyreosis and thyreotoxicosis,

**Table III** Correlation between treatment response at 3 months and changes in the levels of biochemical markers from baseline to the one and three months' evaluations

| | *PR* vs *NC* vs *PD* | *PR* vs *NC* | *NC* vs *PD* | *PR* vs *PD* |
|---|---|---|---|---|
| *n* | 36 | 27 | 27 | 18 |
| ICTP | $r_s$=0.57 (–0.76 to –0.29) | $r_s$=–0.22 (–0.56 to 0.18) | $r_s$=–0.54 (–0.76 to –0.20) | $r_s$=–0.78 (–0.91 to –0.49) |
| PICP | $r_s$=0.37 (–0.63 to –0.04) | $r_s$=–0.07 (–0.45 to 0.33) | $r_s$=–0.46 (–0.72 to –0.09) | $r_s$=–0.48 (–0.77 to –0.02) |
| PIIINP | $r_s$=0.52 (–0.73 to –0.23) | $r_s$=–0.08 (–0.45 to 0.32) | $r_s$=–0.66 (–0.83 to –0.37) | $r_s$=–0.67 (–0.87 to –0.30) |
| sCa | $r_s$=0.10 (–0.24 to 0.42) | $r_s$=0.21 | $r_s$=–0.06 | $r_s$=0.14 |
| uCa | $r_s$=0.29 (–0.04 to 0.57) | $r_s$=0.26 | $r_s$=0.14 | $r_s$=0.40 |
| OHP | $r_s$= –0.52 (–0.73 to –0.23) | $r_s$=–0.38 (–0.66 to 0.00) | $r_s$=–0.33 (–0.63 to 0.06) | $r_s$=–0.72 (–0.89 to –0.38) |
| AP | $r_s$= –0.52 (–0.73 to –0.23) | $r_s$=–0.61 (–0.80 to –0.30) | $r_s$=–0.17 (–0.52 to 0.23) | $r_s$=–0.65 (–0.86 to –0.26) |
| Gla | $r_s$= –0.25 (–0.55 to 0.10) | $r_s$=–0.20 | $r_s$=–0.18 | $r_s$=–0.31 |

$r_s$, Spearman's rank-order correlation coefficient (95% CI in parentheses, for the pairwise comparisons confidence limits are given only when the correlation in the whole material is significant); PR *vs* NC *vs* PD, correlation between marker change (value at 3 months' evaluation divided by baseline value) and treatment response coded as: PD=0, NC=1, PR=2 with all patients included; ICTP, pyridinoline cross-linked carboxy-terminal telopeptide of type I collagen; PICP, carboxy-terminal propeptide of type I procollagen; PIIINP, amino-terminal propeptide of type III procollagen; sCa, serum calcium; uCa, fasting urinary calcium/creatinine; OHP, urinary hydroxyproline/creatinine; AP, serum alkaline phosphatase; Gla; bone gla protein. One patient with NC response had missing values of ICTP, PICP and PIIINP and three patients had missing values of gla (two patients with NC and one with PD).

**Figure 1** Individual concentrations of ICTP and PICP in serum grouped according to treatment response. The stippled lines show the reference interval.

corticosteroid treatment, Paget's disease, osteoporosis, growth hormone deficiency and rheumatoid arthritis (Charles *et al.*, 1994; De la Piedra *et al.*, 1994; Eriksen *et al.*, 1993; Filipponi *et al.*, 1994; Frevert *et al.*, 1994; Hakala *et al.*, 1993; Kerstjens *et al.*, 1994; Sartorio *et al.*, 1993*a*, *b*; Valimaki *et al.*, 1994). In malignant disease of the skeleton high values of this metabolite have been found in multiple myeloma and prostate carcinoma metastatic to the skeleton (Abildgaard *et al.*, 1994; Elomaa *et al.*, 1992; Kylmälä *et al.*, 1993). Although the bone metastases in prostatic carcinoma tend to be osteoblastic rather than lytic, histomorphometric investigations have demonstrated that presence of prostatic cancer cells in the bone leads to both increased degradation and synthesis of bone, even if the net balance often results in increased sclerosis (Taube *et al.*, 1994). PICP, which reflects synthesis of type I collagen is low or normal in multiple myeloma, but often high in prostatic carcinoma metastatic to the skeleton (Elomaa *et al.*, 1992; Kylmälä *et al.*, 1993).

In the present study, the metabolites of type I collagen breakdown and synthesis tended to increase in concert, reflecting the fact that breast cancer cells in the bone marrow simultaneously increased both the synthesis and degradation of bone. Both ICTP and PICP also showed significant positive correlations with both hydroxyproline excretion in the urine and the activity of alkaline phosphatase in serum. In this respect bone metastases from breast cancer resemble prostatic carcinoma more than multiple myeloma. The frequency of high PICP values, however, was only 24% in this study, compared to about 60% in a study of prostatic carcinoma (Kylmälä *et al.*, 1993), probably reflecting the lower bone formation activity in bone metastases from breast carcinoma.

Surprisingly the correlation between collagen metabolism and urinary calcium excretion was negative. It is possible that humoral mediators excreted by mammary carcinoma cells e.g. PTHrp, a peptide inhibiting the excretion of calcium in the urine, modify calcium handling by the kidneys (Bonjour *et al.*, 1988), making calcium excretion an unreliable indicator of skeletal metabolism in this disease. If this is the case, however, serum calcium should increase more with increased bone resorption than if calcium handling by the kidneys were unaffected by the carcinoma. In fact there was no correlation between serum calcium and the indicators of type I collagen metabolism. Thus, the most probable explanation for the failure of calcium excretion to behave as a marker of metastatic activity in the skeleton is that the coupling of bone resorption and formation was retained with a slight surplus of bone formation in most patients. As urinary calcium excretion reflects the balance between bone mineralisation and resorption rather than skeletal turnover, it is conceivable that it should be a poor marker of metastatic activity in the skeleton. The same consideration should also hold for serum calcium, which as expected showed no correlation with treatment response or type I collagen turnover.

Both ICTP and PICP showed a positive correlation with metastatic activity, as reflected by the association between these markers and the number of skeletal metastases and by significant correlations with the treatment response. ICTP seemed to be the more reliable indicator of the two metabolites, as expected, since bone lysis should be more closely associated with collagen breakdown than with its synthesis. The diagnostic accuracy of both ICTP and PICP in assessing the treatment response was at the same level as published results on classical tumour markers Ca 15-3 and CEA (Robertson *et al.*, 1991).

Most biochemical markers behaved similarly in responding and stable patients. These two groups on the other hand, differed significantly from the progressors with respect to the changes in ICTP and PICP levels. The only marker that showed any difference between a partial response and a stable disease was AP, which seemed to decrease more in the responding than in the stable patients. The inability of the type I collagen metabolites to distinguish between responding and stable patients should not be seen as a major drawback, since patients with stable skeletal metastases from mammary carcinoma have as favourable a prognosis as the responding patients (Bitran *et al.*, 1980; Blomqvist *et al.*, 1987), and should probably be managed as responders.

In a previous study on the same patient material we have reported that PIIINP, a metabolite reflecting the synthesis of type III collagen, also correlated with metastatic activity (Blomqvist *et al.*, 1987). There was in fact a significant correlation between the concentrations of PIIINP and those of both ICTP and PICP. PIIINP is a peptide formed during the synthesis of type III collagen, which occurs in most non-mineralised connective tissues together with type I collagen. High values of PIIINP have been found in gynaecological malignancies (Kauppila *et al.*, 1989; Tomas *et al.*, 1990, 1991), multiple myeloma (Taube *et al.*, 1992), and soft-tissue sarcomas (Wiklund *et al.*, 1992, 1993) as well as in a variety of non-malignant disorders like rheumatoid arthritis

(Eberhardt et al., 1990), hepatitis and liver cirrhosis (Niemelä et al., 1990) and myelofibrosis (Hasselbalch et al., 1990). The exact mechanism for the raised values of PIIINP in malignant disease has not yet been clarified. Type III collagen is, however, found in the reticular fibres of the bone marrow, and the marrow in multiple myeloma has been shown to contain large amounts of type III collagen (Taube et al., 1992), suggesting one potential source of PIIINP in skeletal metastatic disease.

One limitation of this study was that the biochemical marker levels were monitored only during a 3 months time window during the treatment. No values were available from the start of systemic treatment, since the inclusion criteria of the trial demanded stable systemic treatment for at least 3 months from the start to 3 months following randomisation. The results of this study therefore have no relevance for the early detection of a treatment response. Theoretically, the biochemical markers could behave differently during the first few months of treatment. In a study by Coombes et al. on the value of various markers for the assessment of a treatment response in skeletal metastases from breast cancer, 45% of the patients responding to the treatment showed an increase in urinary hydroxyproline excretion at the 2–4 months assessment compared with only 11% 6–8 months after the start of treatment. The initial rise in hydroxyproline excretion may be a biochemical equivalent to the tumour flare occasionally seen in responding patients on bone scans (Coombes et al., 1983). Further studies should be done to clarify whether ICTP and PICP are less reliable for the assessment of treatment response within the first few months of treatment. Another weakness of this study was the heterogeneity in the treatment schedules given to the patients, which makes it impossible to study potential interactions between marker levels and treatment itself. It has been shown previously (Wiklund et al., 1993) that chemotherapy per se increases type III collagen turnover and PIIINP. The effect of chemotherapy on the PIIINP level was, however, relatively small, on average a 13% increase during 100 days of treatment. Whether chemotherapy has an impact on ICTP and PICP levels is presently unknown.

Metabolites of type I collagen degradation and biosynthesis, in particular the degradation marker ICTP, seem to correlate with disease activity of breast cancer metastatic to the skeleton. Thus, assessment of metabolism of type I collagen could be an alternative to the use of conventional tumour markers in the monitoring of skeletal metastases from breast carcinoma.

### References

ABILDGAARD N, NIELSEN JL AND HEICKENDORFF L. (1994). Connective tissue components in serum in multiple myeloma: analyses of propeptides of type I and type III procollagens, type I collagen telopeptide, and hyaluronan. *Am. J. Hematol.*, **46**, 173–178.

BITRAN JD, BEKDERMAN C AND DESSER RK. (1980). The predictive value of serial bone scans in assessing response to chemotherapy in advanced breast cancer. *Cancer*, **45**, 1562–1568.

BLOMQVIST C, ELOMAA I, VIRKKUNEN P, PORKKA L, KARONEN S-L, RISTELI L AND RISTELI J. (1987). The response evaluation of bone metastases in mammary carcinoma. The value of radiology, scintigraphy and biochemical markers of bone metabolism. *Cancer*, **60**, 2907–2912.

BLOMQVIST C, ELOMAA I, PORKKA L, KARONEN S-L AND LAMBERG-ALLARDT C. (1988). Evaluation of salmon calcitonin treatment in bone metastases from breast cancer. A controlled trial. *Bone*, **9**, 45–51.

BONJOUR JP, PHILIPPE J AND GUELPA G. (1988). Bone and renal components in hypercalcemia of malignancy and responses to a single infusion of clodronate. *Bone*, **9**, 123–130.

CHARLES P, MOSEKILDE L, RISTELI L, RISTELI J AND ERIKSEN EF. (1994). Assessment of bone remodeling using biochemical indicators of type I collagen synthesis and degradation: relation to calcium kinetics. *Bone Miner.*, **24**, 81–94.

COOMBES RC, DADY P, PARSONS C, McCREADY VR, FORD HT, GAZET J-C AND POWLES TJ.(1983). Assessment of response of bone metastases to systemic treatment in patients with breast cancer. *Cancer*, **52**, 610–614.

De la PIEDRA C, DIAZ MM, DIAZ DE, LOPEZ GE, GONZALEZ PE, CARAMELO C AND RAPADO A. (1994). Serum concentrations of carboxyterminal cross-linked telopeptide of type I collagen (ICTP), serum tartrate resistant acid phosphatase, and serum levels of intact parathyroid hormone in parathyroid hyperfunction. *Scand. J. Clin. Lab. Invest.*, **54**, 11–15.

EBERHARDT K, THORBJORN JL, HORSLEV PK, PETTERSSON H, LORENZEN I AND WOLLHEIM F. (1990). Serum aminoterminal type III procollagen peptide in early rheumatoid arthritis: relation to disease activity and progression of joint damage. *Clin. Exp. Rheumatol.*, **8**, 335–340.

ELOMAA I, VIRKKUNEN P, RISTELI L AND RISTELI J. (1992). Serum concentration of the cross-linked carboxyterminal telopeptide of type I collagen (ICTP) is a useful prognostic indicator in multiple myeloma. *Br. J. Cancer*, **66**, 337–341.

ERIKSEN EF, CHARLES P, MELSEN F, MOSEKILDE L, RISTELI L AND RISTELI J. (1993). Serum markers of type I collagen formation and degradation in metabolic bone disease: correlation with bone histomorphometry. *J. Bone Miner. Res.*, **8**, 127–132.

FILIPPONI P, PEDETTI M, BEGHE F, GIOVAGNINI B, MIAM M AND CRISTALLINI S. (1994). Effects of two different bisphosphonates on Paget's disease of bone: ICTP assessed. *Bone*, **15**, 261–267.

FREVERT EU, BIESTER A, MULLER MJ, SCHMIDT GH, VON ZUR MUHLEN A AND BRABANT G. (1994). Markers of bone metabolism during short-term administration of thyroxine in healthy volunteers. *Eur. J. Endocrinol.*, **131**, 145–149.

GARDNER MJ AND ALTMAN DG. (1989). *Statistics with Confidence*. British Medical Journal: London.

GARDNER SB, WINTER PD AND GARDNER MJ. (1989). *CIA version 1.0*. MJ Gardner and British Medical Journal: London.

HAKALA M, RISTELI L, MANELIUS J, NIEMINEN P AND RISTELI J. (1993). Increased type I collagen degradation correlates with disease severity in rheumatoid arthritis. *Ann. Rheum. Dis.*, **52**, 866–869.

HASSELBALCH H, JUNKER P, HORSLEV PK, LISSE I AND BENTSEN KD. (1990). Procollagen type III aminoterminal peptide in serum in idiopathic myelofibrosis and allied conditions: relation to disease activity and effect of chemotherapy. *Am. J. Hematol.*, **33**, 18–26.

HAYWARD JL, RUBENS R, CARBONE PP, HEUSON J-C, KUMAOKA S AND SEGALOF FA. (1977). Assessment of response to therapy in advanced breast cancer. *Br. J. Cancer*, **35**, 292–298.

KAUPPILA A, PUISTOLA U, RISTELI J AND RISTELI L. (1989). Amino-terminal propeptide of type III procollagen: a new prognosis indicator in human ovarian cancer. *Cancer Res.*, **49**, 1885–1889.

KERSTJENS HA, POSTMA DS, VAN DOORMAAL JJ, VAN ZANTEN AK, BRAND PL, DEKHUIJZEN PN AND KOETER GH. (1994). Effects of short-term and long-term treatment with inhaled corticosteroids on bone metabolism in patients with airways obstruction. Dutch CNSLD Study Group. *Thorax*, **49**, 652–656.

KYLMÄLÄ T, TAMMELA T, RISTELI L, RISTELI J, TAUBE T AND ELOMAA I. (1993). Evaluation of the effect of oral clodronate on skeletal metastases with type I collagen metabolites. A controlled trial of the Finnish Prostate Cancer Group. *Eur. J. Cancer*, **29A**, 821–825.

MELKKO J, NIEMI S, RISTELI L AND RISTELI J. (1990). Radioimmunoassay of the carboxyterminal propeptide of human type I procollagen. *Clin. Chem.*, **36**, 1328–1332.

NIEMELÄ O, RISTELI J, BLAKE JE, RISTELI L, COMPTON KV AND ORREGO H. (1990). Markers of fibrogenesis and basement membrane formation in alcoholic liver disease: relation to severity, presence of hepatitis, and alcohol intake. *Gastroenterology*, **98**, 1612–1619.

PRICE PA AND NISHIMOTO SK. (1980). Radioimmunoassay for the vitamin-K dependent protein of bone and its discovery in plasma. *Proc. Natl Acad. Sci. USA*, **77**, 2234–2238.

RISTELI J, ELOMAA I, NIEMI S, NOVAMO A AND RISTELI L. (1993). Radioimmunoassay for the pyridinoline cross-linked carboxyterminal telopeptide of type I collagen: a new serum marker of bone collagen degradation. *Clin. Chem.*, **39**, 635–640.

ROBERTSON JF, PEARSON D, PRICE MR, SELBY C, BLAMEY RW AND HOWELL A. (1991). Objective measurement of therapeutic response in breast cancer using tumour markers. *Br. J. Cancer*, **64,** 757–763.

SARTORIO A, CONTI A AND MONZANI M. (1993*a*). New markers of bone and collagen turnover in children and adults with growth hormone deficiency. *Postgrad. Med. J.*, **69,** 846–850.

SARTORIO A, CONTI A, MONZANI M, MORABITO F AND FAGLIA G. (1993*b*). Growth hormone treatment in adults with GH deficiency: effects on new biochemical markers of bone and collagen turnover. *J. Endocrinol. Invest.*, **16,** 893–898.

STATXACT. (1992). *Version 2.11.* Cytel Software Corporation: Cambridge, MA.

TAUBE T, FRANSSILA K, RISTELI L, RISTELI J AND ELOMAA I. (1992). Monitoring of multiple myeloma and bone marrow fibrosis with aminoterminal propeptide of type III collagen (PIIINP). *Br. J. Haematol.*, **82,** 32–37.

TAUBE T, KYLMÄLÄ TC, LAMBERQ-ALLARDT C, TAMMELA TLJ AND ELOMAA I. (1994). The effect of clodronate on bone in metastatic prostate cancer. Histomorphometric report of a double blind randomised placebo controlled study. *Eur. J. Cancer*, **30,** 751–758.

TOMAS C, PENTTINEN J, RISTELI J, RISTELI L, VUORI J AND KAUPPILA A. (1990). Serum concentrations of CA 125 and aminoterminal propeptide of type III procollagen (PIIINP) in patients with endometrial carcinoma. *Cancer*, **66,** 2399–2406.

TOMAS C, RISTELI J, RISTELI L, VUORI J AND KAUPPILA A. (1991). Use of various epithelial tumor markers and a stromal marker in the assessment of cervical carcinoma. *Obstet. Gynecol.*, **77,** 566–572.

VALIMAKI MJ, TAHTELA R, JONES JD, PETERSON JM AND RIGGS BL. (1994). Bone resorption in healthy and osteoporotic postmenopausal women: comparison markers for serum carboxy-terminal telopeptide of type I collagen and urinary pyridinium cross-links. *Eur. J. Endocrinol.*, **131,** 258–262.

WIKLUND TA, ELOMAA I, BLOMQVIST CP, RISTELI L AND RISTELI J. (1992). Type III collagen metabolism in soft tissue sarcomas. *Br. J. Cancer*, **65,** 193–196.

WIKLUND TA, BLOMQVIST CP, RISTELI L, RISTELI J AND ELOMAA I. (1993). Impact of chemotherapy on collagen metabolism: a study of serum PIIINP (aminoterminal propeptide of type III procollagen) in advanced sarcomas. *J. Cancer Res. Clin. Oncol.*, **119,** 160–164.